# Characterising the Phenotypic Diversity of Antigen-Specific Memory B Cells Before and After Vaccination

**DOI:** 10.3389/fimmu.2021.738123

**Published:** 2021-09-28

**Authors:** M. Christian Tjiam, Sonia Fernandez, Martyn A. French

**Affiliations:** ^1^ School of Biomedical Sciences, The University of Western Australia, Crawley, WA, Australia; ^2^ Division of Immunology, PathWest Laboratory Medicine, Nedlands, WA, Australia

**Keywords:** antigen-specific B cell, vaccine, isotype, IgD, IgM, IgA, IgG, B-cells

## Abstract

The diversity of B cell subsets and their contribution to vaccine-induced immunity in humans are not well elucidated but hold important implications for rational vaccine design. Prior studies demonstrate that B cell subsets distinguished by immunoglobulin (Ig) isotype expression exhibit divergent activation-induced fates. Here, the antigen-specific B cell response to tetanus toxoid (TTd) booster vaccination was examined in healthy adults, using a dual-TTd tetramer staining flow cytometry protocol. Unsupervised analyses of the data revealed that prior to vaccination, IgM-expressing CD27^+^ B cells accounted for the majority of TTd-binding B cells. 7 days following vaccination, there was an acute expansion of TTd-binding plasmablasts (PB) predominantly expressing IgG, and a minority expressing IgA or IgM. Frequencies of all PB subsets returned to baseline at days 14 and 21. TTd-binding IgG^+^ and IgA^+^ memory B cells (MBC) exhibited a steady and delayed maximal expansion compared to PB, peaking in frequencies at day 14. In contrast, the number of TTd-binding IgM^+^IgD^+^CD27^+^ B cells and IgM-only CD27^+^ B cells remain unchanged following vaccination. To examine TTd-binding capacity of IgG^+^ MBC and IgM^+^IgD^+^CD27^+^ B cells, surface TTd-tetramer was normalised to expression of the B cell receptor-associated CD79b subunit. CD79b-normalised TTd binding increased in IgG^+^ MBC, but remained unchanged in IgM^+^IgD^+^CD27^+^ B cells, and correlated with the functional affinity index of plasma TTd-specific IgG antibodies, following vaccination. Finally, frequencies of activated (PD-1^+^ICOS^+^) circulating follicular helper T cells (cT_FH_), particularly of the CXCR3^-^CCR6^-^ cT_FH_2 cell phenotype, at their peak expansion, strongly predicted antigen-binding capacity of IgG^+^ MBC. These data highlight the phenotypic and functional diversity of the B cell memory compartment, in their temporal kinetics, antigen-binding capacities and association with cT_FH_ cells, and are important parameters for consideration in assessing vaccine-induced immune responses.

## Introduction

Long-term vaccine efficacy is established by generating a pool of antigen-experienced memory lymphocytes that are primed to react faster, with greater intensity, and in the case of B cells, with the ability to bind antigen with higher affinity than in the primary immune response. Memory B cells (MBC) and antibody (Ab)-secreting plasma cells (PC)/plasmablasts (PB) are the B cell-lineages responsible for mediating vaccine-specific humoral immunity. Long-lived PC reside in bone marrow stromal cell niches where they receive survival factors that allow secretion of Abs for decades (1). In contrast, MBC circulate quiescently in blood and lymphoid tissue and constitute a reserve of cells capable of differentiating into PCs that produce affinity-matured Abs. Upon re-challenge, MBC migrate to subcapsular proliferative foci (SPF) in lymphoid tissue where they interact with memory follicular helper T (T_FH_) cells and undergo PB differentiation with heightened kinetics and magnitude thereby boosting antigen-specific Ab production (2). A proportion of re-activated MBC also seed germinal centres (GC) (3, 4) where they undergo somatic hypermutation (SHM) of their immunoglobulin (Ig) genes and are selected for expression of B cell receptors (BCRs) with high affinity for antigens by a limited number of T_FH_ cells (5).

In humans, CD27 has been used as a marker of MBC as the CD27^+^ B cell compartment is enriched for cells that exhibit rapid activation kinetics compared to naïve B cells (6), possesses SHMs in Ig genes (7) (indicating a post-GC history) and constitutes the majority of isotype-switched B cells. However, approximately 50% of CD27^+^ B cells in blood are unswitched IgM-expressing subsets (7) that include IgM^+^IgD^+^ and IgM^+^IgD^lo^ (“IgM-only”) populations, which are transcriptionally more similar to IgG^+^ MBC than to naïve B cells (8). While IgG^+^ and IgA^+^ MBC are generated following GC reactions, the origin(s) of IgM^+^CD27^+^ B cells are less well defined. IgM-only CD27^+^ B cells are proposed to be post-GC B cells as these cells are absent in individuals with congenital defects of GC formation (9). In contrast, the derivation of IgM^+^IgD^+^CD27^+^ B cells, which are the predominant IgM^+^CD27^+^ B cell population in blood, remains unclear. At least a proportion of IgM^+^IgD^+^CD27^+^ B cells are GC-derived because this subset is reduced in CD40 ligand-deficient individuals (10) and somatic mutations in B-cell lymphoma protein 6 (Bcl-6), acquired when Bcl-6 and activation-induced cytidine deaminase are co-expressed in the GC, are present in a proportion of IgM^+^IgD^+^CD27^+^ B cells (11, 12). Toll-like receptors (TLRs) may also be involved in the maintenance and/or generation of IgM^+^IgD^+^CD27^+^ B cells as genetic defects in myeloid differentiation primary response 88, interleukin-1 receptor-associated kinase 4 and Toll/interleukin-1 receptor domain-containing adapter protein, which transduce signalling downstream of TLRs, result in significantly lower circulating numbers of IgM^+^IgD^+^CD27^+^ B cells (13). Recently, it has been proposed that the IgM^hi^ subpopulation of IgM^+^IgD^+^CD27^+^ B cells represents circulating marginal zone-like B cells (14). The biological implications of having such a heterogeneous CD27^+^ B cell compartment are still unfolding. A small minority of IgG^+^ and IgA^+^ MBC that lack CD27 also exist (9) and exhibit low levels of SHM. IgG^+^CD27^-^ MBC are enriched for ‘upstream’ IgG3 and IgG1 subclasses (9, 15, 16), have limited replication history (9) and may therefore constitute early products of GCs. IgA^+^CD27^-^ MBC share molecular similarities with IgA^+^ B cells found in gut lamina propria that likely arise from T-independent responses (9). Alternatively, switched CD27^-^ B cells may derive from extrafollicular (EF) pathways of differentiation, which are notably expanded in systemic lupus erythematosus (17) and severe coronavirus disease-19 (18).

While IgM^+^CD27^+^ B cells exhibit characteristics of MBC, including somatically mutated Ig (7) and Bcl-6 genes (11, 12) acquired in GC reactions, heightened responsiveness to stimulation (19) and transcriptional similarity with IgG^+^ MBC (8), they also exhibit functional differences to classical switched MBC. Akin to marginal zone B cells, IgM^+^CD27^+^ B cells may serve important roles as ‘first responders’, as these cells are capable of chemotaxis and isotype switching in response to neutrophil-derived signals, including β2-adrenoceptor agonists and interferon-γ (8). Compared to IgG^+^ MBC, IgM^+^CD27^+^ B cells are more chemotactically responsive to the GC-derived CXC-motif chemokine ligand 13 (CXCL13) and upregulate Bcl-6 upon BCR stimulation, indicating a GC-biased activation fate (8). In contrast, IgG^+^ MBC are primed for plasma cell differentiation (3, 4, 8), which may be due to a combination of an inherently effective signalling capacity of the IgG BCR (20, 21) and low expression of repressors of plasma cell differentiation Forkhead Box P1 (22) and BTB Domain and CNC Homolog 2 (23). In mice, the IgG1^+^ MBC subset is enriched for cells exhibiting the co-stimulation-primed CD80^+^PD-L2^+^CD73^+^ phenotype (24). The enhanced differentiation propensity of IgG^+^ MBC may also affect their long-term stability. In a mouse model of phycoerythrin (PE) immunisation, Pape et al. reported that PE-specific IgM^+^ MBC were longer-lived than PE-specific IgG^+^ MBC (4). In part, effector differentiation propensity may be explained by molecular characteristics of the IgG BCR that allow for an intrinsically high burst-forming capacity (25). However, BCR affinity for antigens is also a likely factor that contributes to the effector differentiation potential of IgG^+^ MBC, because high BCR affinity reduces the threshold of B cell activation (26). The notion of BCR affinity as a factor that may affect MBC longevity is supported by another study conducted by Pape et al. that demonstrated that BCR affinity was inversely associated with MBC half-life, irrespective of BCR Ig isotype (27). Of importance to MBC affinity maturation is the T_FH_ cell subset of CD4^+^ T cells which reside in limited numbers in SPF of lymphoid tissues (2) and GC (5) and provide selective help to B cells capable of binding antigen with high affinity (5). Despite recognition of the divergent nature of switched and unswitched MBC, the contribution of MBC defined by BCR Ig isotype, and factors that may regulate their longevity, to vaccine-specific Ab responses are not well studied in humans.

To gain insight into the B cell subsets involved in memory and recall responses to vaccines, we studied the B cell and antibody response to tetanus toxoid (TTd), as a model of a prototypical T-dependent antigen, in healthy adults before and after immunisation with adult diphtheria tetanus toxoid (ADT) vaccine. Here, we show that human TTd-specific B cells are diverse; distinguished not only by BCR Ig isotype but also by pre- and post-vaccination frequencies, expansion dynamics, maturation of affinity and associations with frequencies of circulating T_FH_ (cT_FH_) cells, particularly the cT_FH_2 cell subpopulation. Our data suggest that while the generation of IgG^+^ MBC and PB with high affinity BCRs is a hallmark of the effector response, the immune system retains a pool of stable, low-affinity IgM^+^CD27^+^ B cells. Furthermore, we demonstrate, that cT_FH_ cell expansion after vaccination is a strong predictor of BCR affinity maturation amongst IgG^+^ MBC, but not of their clonal expansion. These findings enhance our understanding of the mechanisms that underlie the generation of antibody responses and have important implications for the improvement of vaccine efficacy and longevity.

## Results

### Levels and Functional Affinity of TTd-Specific IgG Antibodies in Plasma Increase Following Vaccination

**Figure 1:**
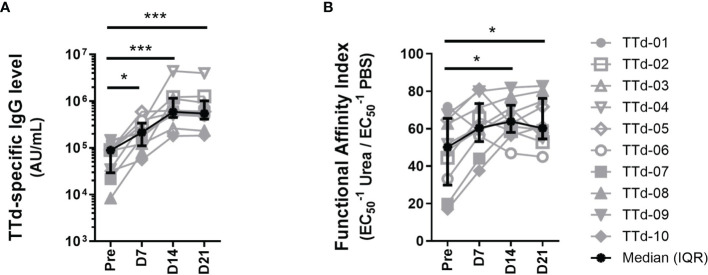
Plasma TTd-specific IgG antibody levels and functional affinity index before and after booster ADT vaccination in healthy adults. **(A)** Plasma TTd-specific IgG antibody levels were measured, in duplicate, using an in-house TTd-specific IgG ELISA. **(B)** The same ELISA was modified to include a chaotropic elution with either 8M urea (or PBS as a control) following the sample incubation step, to elute low affinity antibodies. Functional affinity index was then calculated by expressing the reciprocal half maximal binding (1/EC50) of urea-treated samples as a percentage of that of the PBS control **(B)**. (*, and *** denote p < 0.05, and 0.0005, respectively).

We first investigated the TTd-specific humoral immune response to ADT vaccination in 10 healthy adults. Plasma samples collected before and at 7, 14 and 21 days following ADT vaccination were assayed using an in-house TTd-specific IgG Ab ELISA to measure levels of TTd-specific IgG Abs. Levels of plasma TTd-specific IgG Abs increased following vaccination (p<0.0001; Friedman test) and TTd-specific IgG Ab levels were significantly higher than pre-vaccination levels at days 7 (p<0.05), 14 and 21 post-vaccination (p<0.0005; Dunn’s multiple comparison test; **Figure 1A**).

The same ELISA was modified to include an extra step where each sample was pulsed with the chaotrope urea at a pre-titrated concentration of 8M in order to elute low-affinity IgG Abs. By calculating reciprocal half-maximal binding (1/EC_50_) of 8M urea-pulsed samples as a percentage of the same sample pulsed with PBS, functional affinity of polyclonal IgG Abs can be quantified (28, 29). Functional affinity index (FAI) of plasma TTd-specific IgG Abs increased following vaccination (p = 0.008; **Figure 1B**). FAI of TTd-specific IgG Abs were higher than pre-vaccination levels at days 14 and 21 (p<0.05 for both; **Figure 1B**). Levels of TTd-specific IgG Abs and FAI moderately correlated (r=0.38, p=0.02; Spearman’s correlation). These data show that both Ab level and functional affinity of TTd Abs increased following ADT booster vaccination.

### Dual Tetramer Staining Identifies TTd-Specific B Cells and Plasmablasts

**Figure 2:**
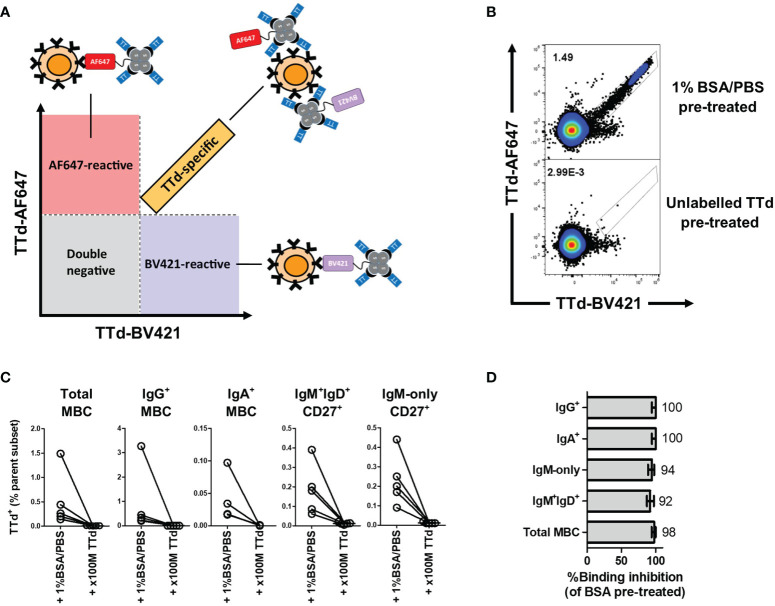
B cell TTd tetramer-binding assay. **(A)** A diagram depicting the theoretical basis of the gating strategy. TTd binding events will bind with equal fluorescence intensities to the tetramer and thus will align proportionally in the double positive quadrant of a TTd-AF647 *vs.* TTd-BV421 biaxial plot. In contrast, single-positive events represent fluorochrome-reactive B cells, as these are events that bind one tetramer and not the other. **(B)** Representative scatterplot showing the effect of unlabelled TTd *versus* 1%BSA/PBS pre-treatment on dual TTd tetramer staining of day 14 post-ADT PBMC sample. **(C)** Pre-treatment with 100M excess of unlabelled TTd prior to addition of TTd tetramers to PBMC of 5 individuals 14 days following ADT vaccination demonstrates a near complete abolishment of TTd-AF647^+^BV421^+^ events across all MBC subsets. **(D)** Median (range) of %TTd binding inhibition upon pre-treatment with unlabelled TTd, relative to 1%BSA/PBS pre-treated control, are shown for each subset.

Having shown that TTd-specific IgG Ab responses were boosted by vaccination, we next sought to determine the phenotypes of TTd-specific B cell subsets elicited following vaccination. To do this, a dual tetramer staining methodology was employed, as also recently described by others (30, 31). TTd was tetramerised with either BV421 or AF647, and subsequently, peripheral blood mononuclear cells (PBMC) were stained with equimolar amounts of each tetramer. TTd-specific B cells were defined as events that bound with proportional fluorescence intensities to each tetramer (**Figure 2A**). In contrast, single positive events are likely B cells that react with the fluorochrome portion of each tetramer rather than to epitopes of TTd and are therefore excluded to enhance the specificity of antigen-specific B cell discrimination (**Figure 2A**). To demonstrate that the dual TTd tetramer staining strategy was highly specific, PBMC samples of five individuals obtained 14 days following vaccination were pre-treated with either unlabelled TTd at a 100 molar excess of the TTd tetramers, or with flow cytometry buffer (FCB; 1% bovine serum albumin (BSA)/phosphate buffered saline (PBS)), for 30 minutes, prior to adding TTd tetramers. Compared to FCB pre-treated controls, pre-treatment with excess unlabelled TTd reduced the median(IQR) frequency of TTd-AF647^+^BV421^+^ events from 0.26(0.97-0.17)% to 0.006(0.01-0.004)% in total MBC, 0.33(1.86-0.22)% to 0(0.002-0)% in IgG^+^ MBC, 0.02(0.07-0.018)% to 0(0.0005-0)% in IgA^+^ MBC, 0.18(0.30-0.18)% to 0.01(0.02-0.008)% in IgM^+^IgD^+^CD27^+^ B cells and 0.20(0.35-0.13)% to 0.01(0.02-0.01)% in IgM-only CD27^+^ B cells (p=0.06 for all; **Figures 2B, C**). Median binding inhibition upon pre-treatment of unlabelled TTd (as % of FCB pre-treated control) was 98% in total MBC, 100% in IgG^+^ MBC, 100% in IgA^+^ MBC, 92% in IgM^+^IgD^+^CD27^+^ B cells, and 94% in IgM-only CD27^+^ B cells (**Figure 2D**). These data confirm that dual TTd-tetramer staining was highly specific.

### Diversity of TTd-Specific Memory B Cells and Plasmablast Phenotypes Defined by BCR Ig Isotype

**Figure 3:**
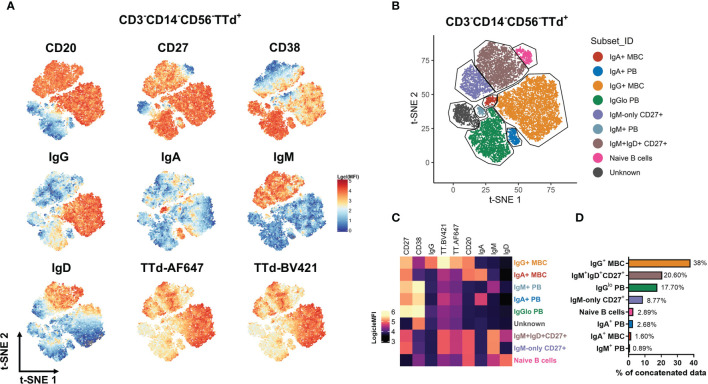
Definition of TTd^+^ B cell subsets by unsupervised analysis using t-SNE. From 3x10^5^ CD3^-^CD14^-^CD56^-^ lymphocytes (n = 40; 10 individuals at 4 timepoints), TTd-BV421^+^AF647^+^ events were exported as individual fcs files. The samples were then concatenated for t-SNE analysis. **(A)** Logicle-transformed MFI was displayed to examine the expression of each marker in the t-SNE dimension-reduced data. **(B)** Subsets were manually gated based on a combination of spatial separation in the t-SNE plot and the differential marker expression. **(C)** For each population identified, surface phenotype is shown on a heatmap. **(D)** Bar graphs denote the percentages of each t-SNE-defined subset in the concatenated data set (all individuals across all timepoints).

To determine the phenotypic diversity of TTd-specific B cell and PB subsets elicited by ADT vaccination, dual TTd tetramer staining was conducted alongside a B cell panel that included Abs to the Ig isotypes: IgM, IgD, IgG and IgA (with exception of IgE as this isotype is directed at responses to helminth parasites and allergens) and subset-defining markers: CD27, CD38 and CD20 that in combination define PBs, memory and naïve B cells. To explore all possible phenotypes of TTd-binding PB and B cell subsets following vaccination, the flow cytometry data was concatenated to undergo unsupervised analysis using t-SNE. Based on the visual expression of each marker (**Figure 3A**) and spatial segregation of clusters within the t-SNE plot, subsets were manually gated (**Figure 3B**). t-SNE analysis revealed eight TTd-binding subsets; three of these subsets were PBs (CD20^-^CD38^+^CD27^++^) expressing either IgM, IgA or IgG^lo^, four subsets were CD20^+^CD27^+^ B cells expressing either IgA, IgG, IgM and IgD or IgM-only and a population of naïve B cells (CD20^+^CD27^-^IgM^+^IgD^+^; **Figure 3C**). In the concatenated data, frequencies of each subset (from most abundant to least abundant) were: IgG^+^ MBC (38%), IgM^+^IgD^+^CD27^+^ B cells (20.60%), IgG^lo^ PB (17.70%), IgM-only CD27^+^ B cells (8.77%), naïve B cells (2.89%), IgA^+^ PB (2.68%), IgA^+^ MBC (1.60%) and IgM^+^ PB (0.89%) (**Figure 3D**). One ‘unknown’ subset that expressed only CD38 was also identified; however, the contribution of this subset to the concatenated data was largely representative of one individual and therefore excluded from subsequent analyses.

### Disparate Expansion Kinetics of Blood TTd-Specific Plasmablasts, Switched and Unswitched B Cell Subsets

**Figure 4:**
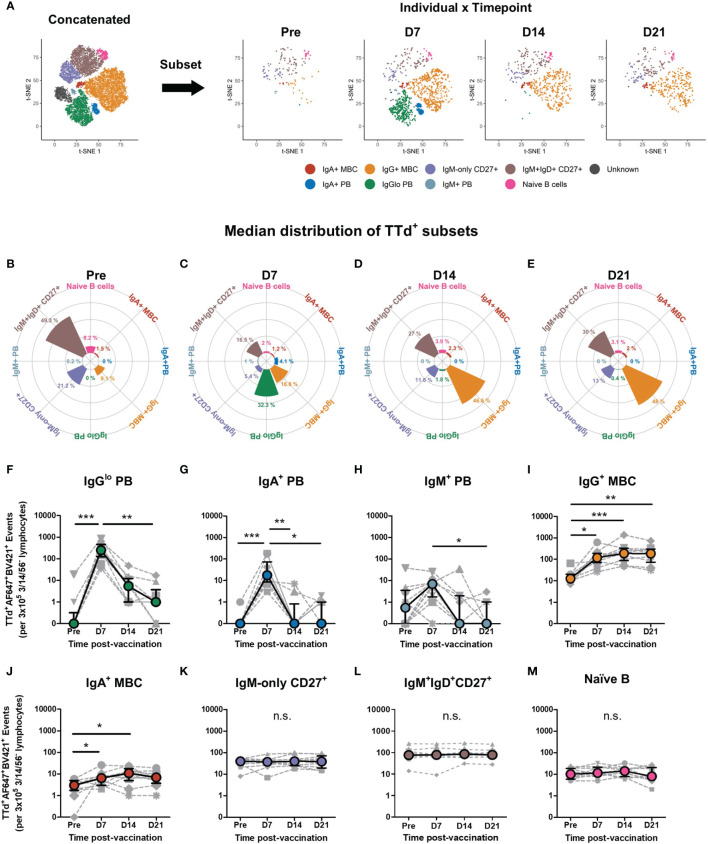
Dynamic changes of TTd^+^ B cell subsets following booster ADT vaccination in healthy adults. **(A)** From the concatenated data representing all TTd^+^ events from 10 individuals at 4 timepoints, the concatenated t-SNE data set underwent data subsetting to examine TTd^+^ events for each individual at each time point (as shown in representative plots of an individual at days 7, 14 and 21 post-ADT). **(B–E)** t-SNE subset distribution (%median proportions) for each timepoint are shown as rose plots. **(F–M)** Median frequencies (events) of each t-SNE-defined subset are shown. (*, ** and *** denote p < 0.05, 0.005 and 0.0005, respectively). n.s., not significant.

The t-SNE analysis identified all TTd-binding B cell subsets present in all samples examined, as the concatenated flow cytometry data consisted of fcs files from all individuals at each timepoint (n=40). To investigate individual vaccine responses and how these B cell subsets changed following vaccination, the concatenated t-SNE dimension-reduced data set underwent data subsetting in order to examine t-SNE data for individual samples at each timepoint (**Figure 4A**). As seen with representative t-SNE plots (**Figure 4A**), it was visually apparent that considerable changes in the TTd-specific B cell compartment occurred within the first three weeks of vaccination. Median proportions of the nine t-SNE-defined B cell subsets in all individuals were examined and changes in median subset distribution (as % of all t-SNE events for the given timepoint) were demonstrated as rose plots for each timepoint (**Figures 4B–E**). Prior to vaccination, median percentages of TTd-binding B cells (from highest to lowest) were: IgM^+^IgD^+^CD27^+^ B cells (49.3%), IgM-only CD27^+^ B cells (21.2%), IgG^+^ MBC (9.1%), naïve B cells (8.2%), IgA^+^ MBC (1.5%), IgM^+^ PB (0.2%), IgA^+^ PB (0%) and IgG^lo^ PB (0%) (**Figure 4B**). At day 7, IgG^lo^ PB were most prevalent (32.3%), followed by IgG^+^ MBC (18.8%), IgM^+^IgD^+^CD27^+^ B cells (16.5%), IgM-only CD27^+^ B cells (5.4%), IgA^+^ PB (4.1%), naïve B cells (2%), IgA^+^ MBC (1.2%) and IgM^+^ PB (1%) (**Figure 4C**). At day 14, IgG^+^ MBC were most abundant (46.6%) followed by IgM^+^IgD^+^CD27^+^ B cells (27.0%), IgM-only CD27^+^ B cells (11.6%), naïve B cells (3.9%), IgA^+^ MBC (2.3%), IgG^lo^ PB (1.8%), IgA^+^ PB and IgM^+^ PB (0% for both) (**Figure 4D**). Subset distribution at day 21 was largely similar to that of day 14, with IgG^+^ MBC as the most frequent subset (48.0%) followed by IgM^+^IgD^+^CD27^+^ B cells (30.0%), IgM-only CD27^+^ B cells (13.0%), naïve B cells (3.1%), IgA^+^ MBC (2.0%), IgG^lo^ PB (0.4%), IgA^+^ PB and IgM^+^ PB (0% for both) (**Figure 4E**).

In addition to examining median distributions of each subset at each timepoint, subset frequencies were also examined as raw event counts (per 3x10^5^ CD3^-^/CD56^-^/CD14^-^ lymphocytes). This is because the t-SNE-defined subsets belong to the same parent data set and thus any percentage change in one subset will reciprocally affect the percentage proportions of remaining subsets. PB subsets exhibited similar transient post-vaccination changes in frequency as previously described (47). TTd^+^IgG^lo^ PB were the most abundant of the PB subsets, and day 7 frequencies were significantly higher than that of pre-ADT vaccination (p < 0.0005) and of day 21 (p<0.005; **Figure 4F**). Similarly, frequencies of TTd^+^IgA^+^ PB were higher at day 7 than pre-ADT (p < 0.0005), day 14 (p < 0.005) and day 21 frequencies (p < 0.05; **Figure 4G**). TTd^+^IgM^+^ PB at day 7 were significantly higher than that of day 21 (p < 0.05; **Figure 4H**). Switched MBC subsets exhibited similar changes in frequencies following vaccination. TTd^+^IgG^+^ MBC frequencies were significantly higher than pre-ADT levels at days 7, 14 and 21 following vaccination (p < 0.05, p < 0.0005, p < 0.005, respectively; **Figure 4I**). TTd^+^IgA^+^ MBC were also higher than pre-ADT levels at day 7 and day 14 following vaccination (p < 0.05 for both; **Figure 4J**). In contrast, TTd^+^IgM^+^IgD^+^CD27^+^ B cells (**Figure 4K**), TTd^+^IgM-only CD27^+^ B cells (**Figure 4L**) and TTd^+^ naïve B cells (**Figure 4M**) exhibited no significant changes in frequency following ADT vaccination.

### Increased TTd Tetramer Binding Capacity After Vaccination Is Observed Amongst IgG^+^ Memory B Cell but Not IgM^+^IgD^+^ B Cells

The ability of BCRs to bind antigen affects the magnitude of downstream signal transduction (26) and thus B cell activation and differentiation propensity. We postulated that the contrasting expansion kinetics observed with switched MBC and unswitched MBC might be related to the antigen binding characteristics of each B cell subset. We aimed to quantify the affinity of TTd binding to BCRs of these distinct populations. One approach for measuring the affinity of BCRs on B cells is to measure the affinity of mAb expressed from sort-isolated B cells, using surface plasmon resonance (SPR). However, this type of analysis is limited to the examination of single clones rather than whole populations and requires soluble (secreted) Ig. Moreover, affinity analyses of soluble Ig exclude the spatial antigen-binding characteristics of membrane-expressed Ig, as BCRs are constrained in a relative planar configuration within the phospholipid bilayer. B cell tetramer binding affinity correlates with the mean fluorescence intensity (MFI) of tetramer binding (32, 33). However, tetramer MFI does not take into account the amount of surface BCR expressed, which likely differs between B cell subsets and individuals. In order to examine antigen binding to B cell populations in different strains of mice, Pape et al. (27) normalised tetramer binding to expression of the BCR-associated β subunit, CD79b, because CD79b is associated to the BCR independent of Ig isotype and with a 1:1 stoichiometry (34, 35). By normalising tetramer MFI to CD79b MFI as a proxy for amount of BCR expressed, this strategy provides an approximation of the average binding affinity of antigen tetramers to BCRs in whole (polyclonal) B cell subsets.

**Figure 5:**
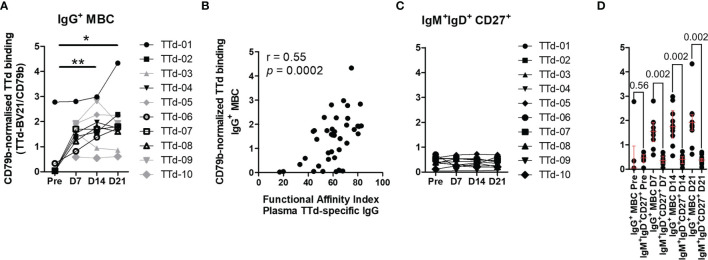
Assessment of CD79b-normalised TTd tetramer binding to B cells before and after booster ADT vaccination. TTd tetramer binding of IgG^+^ MBC and IgM^+^IgD^+^CD27^+^ B cells was assessed relative to expression of the BCR-associated molecule CD79b. **(A)** CD79b-normalised TTd tetramer binding of IgG^+^ MBC (Friedman test, n = 6). Four individuals who had pre-vaccination frequencies of TTd^+^ B cells <10 events per 3x10^5^ lymphocytes were excluded from statistical analyses (shown in grey). **(B)** CD79b-normalised TTd tetramer binding of IgG^+^ MBC was compared to the functional affinity index of plasma TTd-specific IgG antibodies. **(C)** CD79b-normalised TTd tetramer binding of IgM^+^IgD^+^CD27^+^ B cells. **(D)** CD79b-normalised TTd tetramer binding of IgG^+^ and IgM^+^IgD^+^CD27^+^ B cells were compared before and at days 7, 14 and 21 post-ADT (Wilcoxon’s signed rank test). * and ** denote p < 0.05 and p < 0.005, respectively.

We focussed the CD79b-normalised TTd tetramer binding (referred to hereon as ‘CD79b-normalised binding’) analyses on the two most prevalent TTd-binding subsets: IgG^+^ MBC and IgM^+^IgD^+^CD27^+^ B cells. Due to the rare nature of TTd-binding IgG^+^ MBC prior to vaccination, pre-vaccination samples of four individuals that exhibited frequencies less than 10 events per 3x10^5^ cells (an arbitrary cut off that is representative of an event count less than the 10^th^ percentile of TTd^+^IgG^+^MBC^+^ of all samples) were conservatively excluded from the analysis. CD79b-normalised binding increased following ADT vaccination, and was significantly higher than pre-vaccination levels at day 14 (p<0.005) and day 21 (p<0.05; **Figure 5A**). Notably, CD79b-normalised binding of IgG^+^ MBC to TTd correlated with FAI of plasma TTd-specific IgG antibodies following vaccination (r=0.55, p<0.0002; **Figure 5B**). In contrast, CD79b-normalised binding of IgM^+^IgD^+^CD27^+^ B cells to TTd was unchanged following vaccination (**Figure 5C**). Indeed, although CD79b-normalised binding was similar between IgM^+^IgD^+^CD27^+^ B cells and IgG^+^ MBC prior to vaccination, IgG^+^ MBC exhibited higher binding than IgM^+^IgD^+^CD27^+^ B cells at day 7, day 14 and day 21 (p = 0.002, for all; **Figure 5D**).

### PD-1^+^ICOS^+^ Circulating T_FH_ Cell Frequencies at Day 7 Strongly Correlate With Measures of TTd Binding Affinity of IgG^+^ MBC and IgG Antibodies at Day 14 and 21

T_FH_ cells are necessary for B cell affinity maturation and the establishment of PB and MBC subsets. The contrasting expansion dynamics and antigen-binding characteristics of switched and unswitched MBC might therefore be determined by their interactions with T_FH_ cells. We therefore sought to investigate cT_FH_ cells in contemporaneous vaccine samples, to determine frequencies of cT_FH_ cells with an activated phenotype (PD-1^+^ICOS^+^). While *bona fide* T_FH_ cells are lymphoid-resident, there is consensus that blood CD4^+^CXCR5^+^ T cells are a circulating counterpart to lymphoid T_FH_ cells and thus have been studied following administration of influenza (36–40), pneumococcal polysaccharide (41) and HIV-*env*-*gag*-*pol* recombinant adenovirus type 5 vaccines (42), among others (43, 44). Notably, circulating CXCR5^+^ helper T cells exhibit higher B-helper activity than CXCR5^-^ helper T cells, and exhibit transcriptional similarity and clonotypic convergence with tonsillar T_FH_ cells (45).

**Figure 6:**
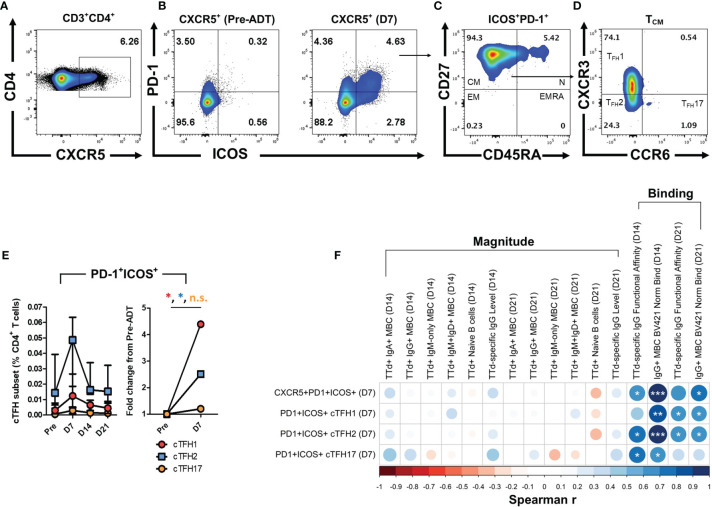
Identification of activated cT_FH_ cells with type 1, 2 and 17 helper T cell phenotypes after booster ADT vaccination and the relationship of day 7 cT_FH_ cell subset frequencies with day 14 and day 21 TTd^+^ B cells and plasma IgG Abs. **(A)** cT_FH_ cells were identified in the CD3^+^CD4^+^ T cell population as CXCR5^+^ cells. **(B)** Within the CXCR5^+^ cT_FH_ cell population, a quadrant gate was applied to identify PD-1^+^ICOS^+^ cT_FH_ cell. Representative plots show an expanded PD-1^+^ICOS^+^ cT_FH_ cell population at day 7. **(C)** Within PD-1^+^ICOS^+^ cT_FH_, cells that exhibited a central memory phenotype (TCM; CD27^+^CD45RA^-^) were further gated. **(D)** Finally, differential CXCR3 and CCR6 expression was used to define the cT_FH_1 (CXCR3^+^CCR6^-^), cT_FH_2 (CXCR3^-^CCR6^-^) and cT_FH_17 (CXCR3^-^CCR6^+^) phenotypes. **(E)** Median (IQR) frequencies (%CD4^+^ T cells) and fold-change of PD-1^+^ICOS^+^ cT_FH_1, cT_FH_2 and cT_FH_17 cells following vaccination. **(F)** A Spearman’s correlation matrix was created to compare frequencies of day 7 PD-1^+^ICOS^+^ cT_FH_ subsets (% of CD4^+^) with the ‘magnitude’ [subset frequencies (events) or Ab levels (AU/ml)] or ‘binding’ parameters (CD79b-normalised TTd binding or plasma IgG TTd^+^ Ab FAI) of t-SNE defined B cell subsets and IgG Abs at days 14 and 21 post-ADT vaccination. *, ** and *** denote p < 0.05, 0.005 and 0.0005, respectively. n.s., not significant.

Activated cT_FH_ cells were identified as CD4^+^ T cells expressing CXCR5^+^ (**Figure 6A**) that were double-positive for PD-1 and ICOS (**Figure 6B**). The majority of cells within the CD4^+^CXCR5^+^PD-1^+^ICOS^+^ population of cells exhibited a central memory T cell phenotype (T_CM_; CD27^+^CD45RA^-^; **Figure 6C**). Within this population, differential expression of CXCR3 and CCR6 was examined to identify cT_FH_ cell subsets that align with type 1 (cT_FH_1; CXCR3^+^CCR6^-^), type 2 (cT_FH_2; CXCR3^-^CCR6^-^) and type 17 (cT_FH_17; CXCR3^-^CCR6^+^) helper T cell phenotypes (**Figure 6D**). PD-1^+^ICOS^+^ cT_FH_ cell subsets transiently expanded 7 days following vaccination, with cT_FH_2 cells being most abundant of the cT_FH_ cell subsets (**Figure 6E**). When examining day 7 fold-change in frequencies of cT_FH_ cell subsets from pre-vaccination levels, PD-1^+^ICOS^+^ cT_FH_1 and cT_FH_2 cell populations exhibited median fold-changes of 4.40 (p = 0.02) and 2.51 (p = 0.05), respectively (**Figure 6E**).

To investigate whether the expansion of PD-1^+^ICOS^+^ cT_FH_ cells at day 7 was associated with parameters of TTd-specific B cells and IgG antibodies at their peak levels (days 14 and 21), Spearman’s correlations matrices were constructed (**Figure 6F**). TTd-specific B cell and IgG antibody measurements were further sub-categorised as measures of ‘magnitude’ (B cell subset frequencies and IgG antibody levels) or ‘binding’ (CD79b-normalised binding of TTd tetramers and plasma IgG antibody FAI). No correlations were observed between frequencies of any PD-1^+^ICOS^+^ cT_FH_ cell subset at day 7 and any measure of MBC or antibody magnitude at day 14 or 21. In contrast, strong correlations were observed between PD-1^+^ICOS^+^ cT_FH_ cells and measures of BCR or antibody affinity (**Figure 6F**). Thus, frequencies of CXCR5^+^PD-1^+^ICOS^+^ cT_FH_ cells at day 7 correlated with the FAI of IgG TTd Abs at day 14 (r = 0.68, p = 0.03) and CD79b-normalised TTd tetramer binding of IgG^+^ MBC at day 14 (r = 0.98, p = 1.47x10^-6^) and day 21 (r = 0.75, p = 0.01). Day 7 frequencies of the PD-1^+^ICOS^+^ cTFH1 cell subset correlated with the CD79b-normalised TTd tetramer binding of IgG^+^ MBC at days 14 and 21 (r = 0.88, p = 8.14x10^-4^ and r = 0.65, p = 0.04, respectively) and the FAI of plasma TTd-specific IgG Abs at day 21 (r = 0.64, p = 0.05). Similarly, day 7 frequencies of PD-1^+^ICOS^+^ cT_FH_2 cells correlated with the FAI of plasma TTd-specific IgG Abs at day 14 and 21 (r = 0.78, p = 0.008 and r = 0.68, p = 0.03, respectively) and CD79b-normalised TTd tetramer binding of IgG^+^ MBC at day 14 and 21 (r = 0.95, p = 2.28x10^-5^ and r = 0.68, p = 0.03, respectively). In addition, day 7 frequencies of PD-1^+^ICOS^+^ cT_FH_17 cells correlated with the FAI of plasma TTd-specific IgG Abs at day 14 (r = 0.73, p = 0.02) and CD79b-normalised TTd tetramer binding of IgG^+^ MBC at day 14 (r = 0.66, p = 0.04).

## Discussion

Cumulative evidence provided by studies in the last decade demonstrate functional distinctions between ‘memory’ subsets of B cells that are defined by expression of different immunoglobulin isotypes in the BCR. A greater understanding of how these B cell subsets contribute to antibody responses will provide strategies for optimising antibody production and facilitate rational vaccine design, particularly for the accelerated development of vaccines against emergent pathogens, such as SARS-CoV-2. To this end, we aimed to characterise antigen-specific B cell subsets that were present in blood before and after vaccination, for which data in the literature reporting this in humans are scarce. Here, dual TTd tetramer staining, with the aid of unsupervised analysis methods, were utilised to explore subsets of blood TTd-specific B cells following ADT vaccination. We demonstrate distinct compartmentalisation of the TTd-specific B cell response. Pre-vaccination, IgM^+^CD27^+^ B cells were the most abundant TTd-binding B cell subset, yet exhibited no observable post-vaccination increases in frequency, in contrast to TTd^+^IgG^+^ MBC, which expanded substantially and peaked at day 14 post-vaccination. Furthermore by employing the method of Pape et al. of normalising tetramer binding to CD79b expression (27), we demonstrate the novel application of this method in humans for examining TTd tetramer binding capacity of B cell subsets and revealed that IgG^+^ MBC, but not IgM^+^IgD^+^CD27^+^ B cells, increased their capacity to bind TTd. Moreover, we demonstrate that this binding capacity correlates with the FAI of plasma IgG TTd antibodies providing evidence that it is a measure of BCR affinity for TTd. Finally, we demonstrate that frequencies of activated (ICOS^+^PD-1^+^) cT_FH_ cell subsets at day 7 predicted TTd binding parameters of IgG^+^ MBC and plasma IgG antibodies at their peak response, but not their frequencies or levels, respectively. These data provide novel insights into the post-vaccination B cell response at the antigen-specific level and highlight the multi-layered nature of the memory response to vaccination.

The data presented here demonstrates that IgG^+^ MBC dominated the TTd-specific B cell response following ADT vaccination. In the concatenated data, IgG^+^ MBC and IgG^lo^ PB together accounted for 55% of TTd-binding B cells. Secondly, it was shown that there were substantial expansions of IgG^+^ subsets at distinct timepoints following vaccination. Similar to other vaccines (36, 46), this included the transient expansion of TTd^+^IgG^lo^ PB at day 7, possibly because PB are trafficking to lymphoid tissues and bone marrow, the principal sites of antibody secretion. In contrast, the expansion dynamics of TTd^+^IgG^+^ MBC was comparatively slower; TTd^+^IgG^+^ MBC initially comprised 9.1% of TTd binding B cells pre-vaccination but increased to become 16.6%, 46.6% and 48.0% of all TTd binding B cells at days 7, 14 and 21, respectively. Finally, in parallel to the post-vaccination increases in frequency of IgG^+^ MBC, examination of CD79b-normalised binding of TTd tetramers revealed that the expanded IgG^+^ MBC subset acquired a steady increase in their capacity to bind TTd. As CD79b-normalised binding capacity of B cells for TTd tetramers correlated with FAI of plasma IgG TTd Abs, we suggest that it may be applicable as a method for assessing the affinity maturation of MBCs in humans, especially after vaccination.

TTd-specific IgA^+^ MBC and PB subsets were also elicited by ADT vaccination with similar kinetics; however, IgA^+^ TTd^+^ subsets represented a minority of the switched B cell response at any given timepoint in comparison to their IgG^+^ counterparts. This is in agreement with the findings of Giesecke et al. (47) who single-cell sorted TTd-specific plasma cells at day 7 and found that the majority of the sorted cells were *Cγ1^+^* and only a minority were *Cα1^+^*. Due to the constraints of our flow cytometry panel we were unable to further discern whether a proportion of these switched cells were activated B cells (CD20^+^CD27^+^CD71^+^Ki-67^+^) committed to the memory lineage (46), CD21^lo^ B cells (CD19^+^CD21^lo^CD27^+^) that are committed to the PC lineage (48), or ‘double negative’ (DN) B cells (CD27^-^IgD^-^CD11c^+^) that have come into prominence as an expanded B cell subset in chronic infection (49) and some autoimmune diseases (17). While TTd vaccination induces primarily ‘classical’ MBC, a small proportion of DN B cells are elicited following TTd vaccination (50), likely as a memory intermediate (50). Furthermore, TTd-binding CD27^-^IgG^+^ and CD27^-^IgA^+^ B cells (9, 15, 16) that may be early products of the GC, or of EF differentiation pathways (17, 18), were not detectable in the post-ADT vaccine samples we analysed. These data suggest that the majority of the switched B cells were the progeny of GC-experienced MBC, as would be expected for a protein booster vaccination.

In contrast to the switched B cell response, TTd-binding IgM^+^CD27^+^ B cell subsets exhibited no change in absolute frequencies following ADT vaccination. Similarly, TTd^+^ naïve B cells, which represent TTd-binding capability of the pre-immune repertoire, exhibited no change in frequencies. Moreover, TTd-binding IgM^+^CD27^+^ B cells were ~10 times more abundant than TTd-binding naïve B cells at any given time point. Despite the lack of an observable increase in post-vaccination frequencies of IgM^+^IgD^+^CD27^+^ and IgM-only CD27^+^ B cells, these subsets accounted for 20.8% and 8.8% of all TTd binding events in the concatenated data and were the most frequent TTd-binding subset prior to ADT vaccination, comprising 49.3% and 21.2% of TTd-binding events, respectively. These results support those described by Della Valle et al. who showed with a limiting dilution assay that the majority of TTd-specific MBC in individuals who had not received a vaccine in the previous 10 years were IgM^+^, whereas IgG^+^ MBC formed a minority of cells (12). Moreover, Della Valle et al. similarly reported that frequencies of TTd-specific IgM^+^CD27^+^ B cells remained unchanged following vaccination, despite exhibiting a molecular footprint consistent with antigen encounter and GC selection (12). V_H_ mutations of TTd-specific IgG^+^ MBC were on average double that of TTd-specific IgM^+^CD27^+^ B cells in immunised individuals (12), which may alone explain the disparity we observed in CD79b-normalised TTd binding between these subsets.

Alternatively, one explanation for the absence of an observable increase in frequencies, or TTd-binding capacity, of TTd^+^ IgM^+^CD27^+^ B cell populations is that their activation results in a GC fate where IgM^+^CD27^+^ B cell undergo isotype switching to become IgG^+^ or IgA^+^ MBC. IgM^+^CD27^+^ B cell subsets possess a GC-skewed fate following activation, including upregulation of Bcl-6, and receptors that favour homing to the GC-derived chemokine CXCL13 upon activation (8). In this instance, the acquisition of a switched phenotype may preclude the ability to detect an expansion of TTd^+^ IgM^+^CD27^+^ B cells following vaccination. Shared clones of TTd-specific MBC have been reported among IgM^+^ and IgM^-^ CD27^+^ B cell subsets (12), demonstrating a derivation of switched MBC from IgM^+^CD27^+^ B cells. In addition, genealogical analyses of peripheral MBC clones show that a proportion of IgG^+^ MBC clones are direct descendants of IgM^+^IgD^+^CD27^+^ B cells (11, 51). However, one may also expect an acute decline in TTd^+^ IgM^+^CD27^+^ B cell frequencies due to switching-induced ‘depletion’ of TTd-specific IgM^+^CD27^+^ B cells, which was not observed. To further investigate this, clonal analyses of TTd-specific IgM^+^CD27^+^ B cells and IgG^+^ MBC at each timepoint would be useful, as this would provide an indication of whether IgM^+^CD27^+^ B cells are recruited to ‘replenish’ the IgG^+^ MBC compartment through switching.

Given these findings, what is the biological significance of a numerically invariant IgM^+^CD27^+^ B cell population, with low capacity to bind antigen? A possible consequence of expressing low-affinity BCRs is a lengthened persistence of ‘low-affinity’ B cell subsets, which is corroborated by our finding that IgM^+^CD27^+^ B cells were the most prevalent subset prior to booster vaccination. Indeed, acquisition of increased BCR affinity is inversely associated with B cell longevity. Thus, mice that possess naïve B cells with germline-encoded BCRs binding with high affinity to PE exhibit an unstable, rapidly depleting PE-specific IgG^+^ MBC population following vaccination (4, 27). In contrast, another strain of mouse with a different set of germline-encoded BCRs that bind with low affinity to PE, exhibit stable PE-specific IgG^+^ MBC (27). Two possible mechanisms have been proposed for the inverse association between BCR affinity and longevity of MBC. First, Gitlin et al. demonstrated that a consequence of the acquisition of SHM/increased affinity was the development of polyreactivity to self-antigens (52). Thus, high affinity MBC clones had an increased probability of clonal deletion due to a higher likelihood of developing SHM-acquired self-reactivity (52). Secondly, higher affinity binding may decrease the threshold for BCR activation (26), and thus high affinity MBC may be depleted at a higher rate than low affinity cells due to a higher propensity for terminal differentiation. Aside from possible enhanced persistence as a result of possessing low-affinity BCRs, low-affinity IgM^+^CD27^+^ B cells may also exist to provide a pool of memory cells with broad antigen reactivity that can counteract immune evasion upon re-infection with antigenic variants of the same pathogen. Support for our proposal that the MBC compartment retains not only antigen binding diversity, but also isotype diversity (ability to generate MBC and PB of all isotypes) in the IgM^+^CD27^+^ B cell subset, is provided by the study of Roco et al. who demonstrated that the output of late GC reactions is primarily IgM^+^ MBCs rather than switched MBCs (53). Therefore, the existence of a long-lived, low-affinity, IgM^+^CD27^+^ B cell subset may be a means of retaining diversity in the MBC compartment.

To further investigate determinants of changes in B cell and antibody responses measured here, cT_FH_ cells with the PD-1^+^ICOS^+^ phenotype, denoting recent activation, were examined. It was observed that PD-1^+^ICOS^+^ cT_FH_ cells exhibited the greatest fold-change from baseline at day 7, namely PD-1^+^ICOS^+^ cT_FH_1 cells (~4.5 fold-increase) followed by PD-1^+^ICOS^+^ cT_FH_2 cells (~2.5 fold-increase). While larger fold-changes from baseline were observed for PD-1^+^ICOS^+^ cT_FH_1 cell frequencies, PD-1^+^ICOS^+^ cT_FH_2 cells were the most frequent cT_FH_ cell subset at any given timepoint. In fact, cT_FH_1 cells are reported to exhibit limited B helper function *in vitro* (54, 55), characterised by lower capacity for CXCL13 production (54), lower IL-21 secretion when co-cultured with naïve B cells (55), lower ability to activate naïve B cells to produce Abs (55), and lower ability to induce PB differentiation of MBC (54), compared to the cT_FH_2 and cT_FH_17 cell subsets. Moreover, at least for quiescent (ICOS^-^PD-1^+^) cT_FH_ cells, the cT_FH_2 and cT_FH_17 cell subsets are the most transcriptionally similar to tonsillar GC T_FH_ cells (24), which may further explain their greater B cell helper ability. The relative abundance and day 7 expansion of PD-1^+^ICOS^+^ cT_FH_2 cells is therefore likely to be a large contributing factor to the effectiveness of TTd vaccination. Influenza vaccination on the other hand, induces a large expansion of predominantly cT_FH_1 cells (36–38, 40). It has been argued by Locci et al. that the expansion of cT_FH_1 cells and not cT_FH_2 cells may underlie the relatively quick decline of protective immunity provided by seasonal influenza vaccination (54). Further data that supports the argument of Locci et al., was the finding that addition of a viral vector to the malaria RTS,S/AS01B vaccine resulted in a cT_FH_1-skewed response, which was associated with reduced Ab production and vaccine efficacy (56). Our data further supports this argument, as ADT vaccination, with an Ab half-life of 11 years (57) and the ability to confer protection with as little as 0.1 IU/mL of vaccine-induced Abs (58), was associated with an overall greater frequency and expansion of PD-1^+^ICOS^+^ cT_FH_2 cells and a smaller expansion of cT_FH_1 cells.

In line with T_FH_ cells being a lineage specialised in the selection of B cells with high affinity BCRs, we showed that PD-1^+^ICOS^+^ cT_FH_ cell subsets at day 7 correlated with plasma TTd-specific IgG Ab functional affinity at their peak levels (days 14 and 21). These findings are consistent with other studies that demonstrate that cT_FH_ cell frequencies at day 7 following trivalent (37) or mRNA (40) influenza vaccination correlate with the affinity of circulating Abs measured with either SPR or chaotropic NaSCN elution. We extend these findings by demonstrating that day 7 frequencies of PD-1^+^ICOS^+^ cT_FH_1 and cT_FH_2 cells significantly, and more strongly, correlated with CD79b-normalised TTd tetramer binding of IgG^+^ MBC at days 14 and 21. However, frequencies of these same cT_FH_ cell subsets at day 7 did not correlate with the frequencies of any TTd-specific MBC subsets, nor TTd-specific IgG Ab levels in plasma, at day 14 and 21. These data suggest that while affinity of both IgG Abs and BCRs on IgG^+^ MBC are directly related to the expansion of cT_FH_ cells, clonal expansion of TTd^+^ IgG^+^ MBC or plasma levels of TTd-specific IgG Abs at later timepoints are not. Our data suggests that examination of PD-1^+^ICOS^+^ cT_FH_ cell frequencies at day 7 after vaccination may be a means of predicting the affinity maturation of both antigen-specific IgG antibodies and IgG^+^ MBC for T-dependent antigens and that this may be a useful parameter for the assessment of novel vaccine candidates.

We acknowledge that our study has potential limitations that must be considered when assessing the significance of the findings. Firstly, an *in vitro* functional comparison of switching kinetics between IgM^+^CD27^+^ B cells and IgG^+^ MBC would have provided informative data to further determine the functional contributions of IgM^+^CD27^+^ B cells to the vaccine-induced response. However, our primary aim was to first characterise the phenotypic diversity of CD27^+^ B cell subsets defined by isotype expression before and after vaccination, and how these subsets related to measures of antibody level, antibody functional affinity and cT_FH_ cell activation. Secondly, our workflow included characterisation of ‘global’ cT_FH_ cell profiles rather than antigen-specific cT_FH_ cell profiles, the latter of which would be optimally measured *via* stimulation with TTd peptide pools in an activation induced marker assay (59), as cT_FH_ cells are limited producers of cytokines as measured by intracellular cytokine staining (60). However, we examined cT_FH_ cells within the expanded, activated ICOS^+^PD-1^+^ subset at day 7 post-vaccination, a population that is virtually absent at other timepoints (Fig 6B, E). Hence, the ‘global’ cT_FH_ cell population examined in this manner is enriched for TTd-specific cT_FH_ cells, as demonstrated by their strong correlations with TTd-specific IgG antibody and IgG^+^ MBC binding parameters at days 14 and 21 following vaccination.

In summary, we demonstrate that antigen-specific B cell and plasmablast subsets are phenotypically and functionally diverse in their post-vaccination expansion dynamics, antigen binding characteristics and their interaction with cT_FH_ cells. In particular, the relative abundance of both IgM^+^CD27^+^ B cells and IgG^+^ MBC may be an important consideration for strategies that seek to optimise vaccine efficacy and/or durability. We propose that the integration of bulk antigen-specific serological measurements, antigen-specific B cells and ICOS^+^PD-1^+^ cT_FH_ cell profiles, as performed here, is a workflow that can be applied to assess the magnitude, quality and durability of antibody responses elicited by vaccines. In this manner, choices of vaccine immunogens, adjuvants and dosing schedules to generate antibody-mediated immunity against pathogens, such as SARS-CoV-2, might be optimised.

## Materials and Methods

### Sample Collection

Lithium heparin anti-coagulated blood was obtained from consenting healthy adults following a routine ADT booster vaccine in accordance with the National Statement on Ethical Conduct in Human Research (2007; updated in 2018), jointly developed by the Australian National Health and Medical Research Council, Australian Research Council and Universities Australia (61). Blood samples were centrifuged against a Ficoll-Hypaque (1.077g/L) density gradient and PBMC were isolated and enumerated under a haemocytometer. PBMC were resuspended at 10^7^ cells/mL in cold freezing medium consisting of 90% heat-inactivated foetal bovine serum and 10% dimethylsulfoxide. PBMC then underwent controlled freezing (Δ -1°C/min to -80°C) before storage in liquid nitrogen.

### Preparation of Fluorescent TTd Tetramers

TTd protein (Statens Serum Institut, DK) was conjugated to biotin *via* amide linkage following reaction of TTd with N-hydroxysuccinimide (NHS) biotin ester (EZ-link sulfo-NHS-LC-biotin labelling kit, ThermoFisher Scientific, Rockford, IL). Excess free biotin was removed with size-exclusion chromatography using Zeba™ 7 kDa molecular weight cut-off desalting columns (ThermoFisher Scientific). The degree of biotinylation was determined to be 6 biotin molecules per molecule of TTd, as measured with the 4’-hydroxyazobenzene-2-carboxylic acid/Avidin biotin quantification kit (ThermoFisher Scientific). Tetramers were created by reacting biotin-TTd with either streptavidin-BV421 (BD Biosciences) or streptavidin-AF647 (BioLegend) at 4:1 molar ratios for 20 mins, on ice, and centrifuged at maximum speed on a benchtop microcentrifuge (18,000 x g) for 10 mins (4°C) to remove aggregates.

### Flow Cytometry Analyses of B Cells and Circulating T_FH_ Cells

PBMC were thawed in PBS (Sigma-Aldrich) and centrifuged at 300 x g for 7 mins. The supernatant was discarded by pipette aspiration. Subsequently, PBMC were resuspended in 1ml of fixable viability stain (FVS) 575V (BD Biosciences, San Jose, CA) diluted 1:1000 in PBS. 1 mL of 2% BSA (AusgeneX, Loganholme QLD)/PBS was added to the tube, centrifuged at 300 x g for 5 mins and the supernatant was discarded. Details of fluorochrome-conjugated antibodies and staining reagents utilised in the three flow cytometry panels in this study are summarised in **Supplementary Table 1**. For examination of TTd-specific B cells, 1x10^7^ PBMC were resuspended in 100μL of FCB consisting of 1%BSA/PBS were stained with the respective Ab cocktail, containing TTd tetramers, for 30 mins at room temperature. For examination of cT_FH_ cells, 5x10^6^ PBMC were resuspended in 100μL of FCB and incubated with the respective Ab cocktail for 15 mins at room temperature. Cells were then washed twice with 2ml of FCB and resuspended in 3ml FCB for acquisition on a 4-laser (405nm; 50mW, 488nm; 50mW, 561nm; 50mW and 638nm; 100mW) Attune NxT acoustic focusing flow cytometer (ThermoFisher Scientific). Where possible, mAb clones were chosen from peer-reviewed optimised multicolour immunofluorescence panels published in *Cytometry Part A (* 62), and titrated to obtain maximal stain indices. A voltage walk was performed using single-stained PBMC to determine photomultiplier tube gain settings that provided greatest signal separation. Acquisition volume was set to 3mL and the flow rate was adjusted to achieve a threshold rate between 2x10^4^ – 2.5x10^4^ events/s. Digital compensation was performed in the Attune NxT software using single stained compensation bead mix (mixture of anti-mouse Ig κ capture beads and unconjugated/negative beads; BD Biosciences).

### Dimensionality Reduction With t-SNE

Flow cytometry data (fcs3.0) files were initially imported into FlowJo v10.4 (FlowJo, LLC, OR). Data acquisition quality was checked with FlowJo QC function and by visual inspection of events using the time parameter. Single cells were identified as cells with proportional FSC-A to FSC-H measurements and lymphocytes were then identified *via* FSC-A/SSC-A profiles. Viable (FVS575V^-^) cells not expressing T cell/natural killer cell/monocyte (CD3^-^CD56^-^CD14^-^) lineage markers were then gated and this population was down-sampled to 3x10^5^ events for each sample. Within down-sampled events, a gate was created at a 45° angle in the double-positive quadrant of BV421 and AF647 channels to identify TTd^+^BV421^+^AF647^+^ cells that bound proportional amounts of each TTd tetramer. TTd^+^BV421^+^AF647^+^ events were exported as individual fcs3.0 files. Each fcs file was then imported into RStudio using the Cytofkit package (63) with the graphical user interface. Fluorescence parameters were logicle (bi-exponentially) transformed and the individual sample files were concatenated for dimensionality reduction analyses. t-distributed stochastic neighbour embedding (t-SNE) was performed on the concatenated data with seed set to 42, perplexity set to 30 and the input fluorescence parameters were: CD27-BV421, CD20-BV510, CD38-APC-H7, IgM-PE-CF594, IgD-AF700, IgA-PE, IgG-PE-Cy7, TTd-BV421 and TTd-AF647. Scatterplots were constructed in RStudio with *ggplot2* and *RColorbrewer* or *ggsci* colour palettes. Heatmaps were constructed in RStudio using *complexheatmaps* and the R implementation of the *viridis* colour palette.

### Measurement of CD79b-Normalised B Cell Binding of TTd Tetramers

Single cells were identified by FSC-H/FSC-A profiles, followed by exclusion of cells expressing FVS575V, CD3, CD14 and CD56. IgG^+^ MBC and IgM^+^IgD^+^CD27^+^ B cells were manually gated. In each of these subsets, TTd^+^BV421^+^AF647^+^ cells were gated and within this gate, the median fluorescence intensities (MdFI) of the TTd (V450/50: BV421) and CD79b (R730/45: APC-R700) channels were exported in an excel file. CD79b-normalised MdFI for each of the populations, for each PBMC sample, was calculated by:


$$Normalised\;surface\;binding=\frac{MdFI\;TTd\;(BV421)}{MdFI\;CD79b\;(APC.R700)}$$

### ELISA to Measure Plasma TTd-Specific IgG Antibody Levels and Avidity

Plasma was collected after centrifugation of lithium-heparin anti-coagulated blood and stored at -80°C. TTd-specific IgG antibodies were measured using an in-house ELISA. Corning half-area 96 well plates were coated with TTd (20μg/ml) overnight at 4°C. The following day, plates were washed with PBS containing 0.05% Tween20 (PBS-T) and blocked with 5% BSA/PBS. Plasma samples were assayed in duplicate, serially diluted in 2%BSA-PBS (assay diluent) for 2hrs at room temperature. Samples were compared to a plasma sample with high levels of TTd-specific IgG antibodies as an arbitrary standard and values were expressed as arbitrary units/ml (AU/ml). The plates were further washed with PBS-T and horseradish peroxidase-conjugated mouse anti-human IgG, diluted 1:4000 in assay diluent, was added to each well for 1hr at room temperature. Plates were washed and 3, 3’, 5, 5’-tetramethylbenzidine substrate was added to initiate a colourimetric reaction. The reaction was terminated with 1M H_2_SO_4_ and the optical density of each well at λ = 450nm, was measured using the SpectraMax microplate reader (Molecular Devices). To measure functional affinity of IgG antibodies to TTd, the TTd ELISA was modified to include an additional incubation following the sample wash step where each sample was pulsed with 8M urea or PBS for 10 minutes at room temperature and the plates were washed to remove eluted antibodies (64, 65). For each sample, a saturation binding curve was generated by plotting OD450 *versus* the logarithm of 1/plasma dilution factor and a three-parameter sigmoidal curve fit was applied in GraphPad Prism (v5.04). The dilution factor required for half-maximal binding (EC_50_) was calculated for urea-treated samples and expressed as a percentage of the PBS-treated control, which yielded the functional affinity index (FAI) (28, 29).


$$Functional\;affinity\;index=\frac{\left(1÷{EC}_{50}\;Urea\;treated\right)}{\left(1÷{EC}_{50}\;PBS\;treated\right)}\times 100$$

### Statistical Analyses

Unless otherwise stated, all statistical tests were undertaken using GraphPad v5.04 and p values < 0.05 were considered statistically significant. Statistical differences between two groups consisting of repeated measures were calculated using Wilcoxon matched pairs signed rank test. For comparisons of repeated measures between three or more groups, Friedman tests were undertaken with Dunn’s multiple comparison *post-hoc* test. Spearman’s correlations were undertaken to examine correlations between groups. Spearman’s correlation matrices were constructed using the *Corrplot* package in R.

## Data Availability Statement

The raw data supporting the conclusions of this article will be made available by the authors, without undue reservation.

## Ethics Statement

Ethical review and approval was not required for the study on human participants in accordance with the local legislation and institutional requirements. Written informed consent for participation was not required for this study in accordance with the national legislation and the institutional requirements.

## Author Contributions

MT performed the laboratory work, data analysis, wrote the manuscript and conceptualised the project. SF supervised laboratory work and the writing of the manuscript. MF supervised laboratory work, writing of the manuscript and conceptualised the project. All authors contributed to the article and approved the submitted version.

## Funding

This work was supported by a Royal Perth Hospital Research Foundation grant 2017/14.

## Conflict of Interest

The authors declare that the research was conducted in the absence of any commercial or financial relationships that could be construed as a potential conflict of interest.

## Publisher’s Note

All claims expressed in this article are solely those of the authors and do not necessarily represent those of their affiliated organizations, or those of the publisher, the editors and the reviewers. Any product that may be evaluated in this article, or claim that may be made by its manufacturer, is not guaranteed or endorsed by the publisher.

## References

[1] Halliley JL Tipton CM Liesveld J Rosenberg AF Darce J Gregoretti IV. Long-Lived Plasma Cells Are Contained Within the CD19– CD38hiCD138+ Subset in Human Bone Marrow. Immunity (2015) 43(1):132–45. doi: 10.1016/j.immuni.2015.06.016 PMC4680845 26187412

[2] Moran I Nguyen A Khoo WH Butt D Bourne K Young C. Memory B Cells Are Reactivated in Subcapsular Proliferative Foci of Lymph Nodes. Nat Commun (2018) 9(1):1–14. doi: 10.1038/s41467-018-05772-7 30135429 PMC6105623

[3] Dogan I Bertocci B Vilmont V Delbos F Mégret J Storck S. Multiple Layers of B Cell Memory With Different Effector Functions. Nat Immunol (2009) 10(12):1292–9. doi: 10.1038/ni.1814 19855380

[4] Pape KA Taylor JJ Maul RW Gearhart PJ Jenkins MK. Different B Cell Populations Mediate Early and Late Memory During an Endogenous Immune Response. Science (2011) 331(6021):1203–7. doi: 10.1126/science.1201730 PMC3993090 21310965

[5] Mesin L Ersching J Victora GD. Germinal Center B Cell Dynamics. Immunity (2016) 45(3):471–82. doi: 10.1016/j.immuni.2016.09.001 PMC5123673 27653600

[6] Tangye SG Avery DT Deenick EK Hodgkin PD. Intrinsic Differences in the Proliferation of Naive and Memory Human B Cells as a Mechanism for Enhanced Secondary Immune Responses. J Immunol (2003) 170(2):686–94. doi: 10.4049/jimmunol.170.2.686 12517929

[7] Klein U Rajewsky K Küppers R. Human Immunoglobulin (Ig) M+ IgD+ Peripheral Blood B Cells Expressing the CD27 Cell Surface Antigen Carry Somatically Mutated Variable Region Genes: CD27 as a General Marker for Somatically Mutated (Memory) B Cells. J Exp Med (1998) 188(9):1679–89. doi: 10.1084/jem.188.9.1679 PMC2212515 9802980

[8] Seifert M Przekopowitz M Taudien S Lollies A Ronge V Drees B. Functional Capacities of Human IgM Memory B Cells in Early Inflammatory Responses and Secondary Germinal Center Reactions. Proc Natl Acad Sci (2015) 112(6):E546–55. doi: 10.1073/pnas.1416276112 PMC4330750 25624468

[9] Berkowska MA Driessen GJ Bikos V Grosserichter-Wagener C Stamatopoulos K Cerutti A. Human Memory B Cells Originate From Three Distinct Germinal Center-Dependent and-Independent Maturation Pathways. Blood (2011) 118(8):2150–8. doi: 10.1182/blood-2011-04-345579 PMC3342861 21690558

[10] Weller S Braun MC Tan BK Rosenwald A Cordier C Conley ME. Human Blood IgM “Memory” B Cells are Circulating Splenic Marginal Zone B Cells Harboring a Prediversified Immunoglobulin Repertoire. Blood (2004) 104(12):3647–54. doi: 10.1182/blood-2004-01-0346 PMC2590648 15191950

[11] Seifert M Küppers R. Molecular Footprints of a Germinal Center Derivation of Human IgM+ (IgD+) CD27+ B Cells and the Dynamics of Memory B Cell Generation. J Exp Med (2009) 206(12):2659–69. doi: 10.1084/jem.20091087 PMC2806629 19917772

[12] Della Valle L Dohmen SE Verhagen OJ Berkowska MA Vidarsson G van der Schoot CE. The Majority of Human Memory B Cells Recognizing RhD and Tetanus Resides in IgM+ B Cells. J Immunol (2014) 193(3):1071–9. doi: 10.4049/jimmunol.1400706 PMC4105240 24965774

[13] Weller S Bonnet M Delagreverie H Israel L Chrabieh M Maródi L. IgM+ IgD+ CD27+ B Cells Are Markedly Reduced in IRAK-4–, MyD88-, and TIRAP-But Not UNC-93B–Deficient Patients. Blood (2012) 120(25):4992–5001. doi: 10.1182/blood-2012-07-440776 23002119 PMC3525023

[14] Bautista D Vásquez C Ayala-Ramírez P Téllez-Sosa J Godoy-Lozano E Martínez-Barnetche J. Differential Expression of IgM and IgD Discriminates Two Subpopulations of Human Circulating IgM+ IgD+ CD27+ B Cells That Differ Phenotypically, Functionally, and Genetically. Front Immunol (2020) 11. doi: 10.3389/fimmu.2020.00736 PMC7219516 32435242

[15] Fecteau JF Côté G Néron S. A New Memory CD27– IgG+ B Cell Population in Peripheral Blood Expressing VH Genes With Low Frequency of Somatic Mutation. J Immunol (2006) 177(6):3728–36. doi: 10.4049/jimmunol.177.6.3728 16951333

[16] De Jong BG IJspeert H Marques L van der Burg M Van Dongen JJ Loos BG. Human IgG2-And IgG4-Expressing Memory B Cells Display Enhanced Molecular and Phenotypic Signs of Maturity and Accumulate With Age. Immunol Cell Biol (2017) 95(9):744–52. doi: 10.1038/icb.2017.43 PMC5636940 28546550

[17] Jenks SA Cashman KS Zumaquero E Marigorta UM Patel AV Wang X. Distinct Effector B Cells Induced by Unregulated Toll-Like Receptor 7 Contribute to Pathogenic Responses in Systemic Lupus Erythematosus. Immunity (2018) 49(4):725–39.e726. doi: 10.1016/j.immuni.2018.08.015 30314758 PMC6217820

[18] Woodruff MC Ramonell RP Nguyen DC Cashman KS Saini AS Haddad NS. Extrafollicular B Cell Responses Correlate With Neutralizing Antibodies and Morbidity in COVID-19. Nat Immunol (2020) 21(12):1506–16. doi: 10.1038/s41590-020-00814-z PMC7739702 33028979

[19] Shi Y Agematsu K Ochs HD Sugane K. Functional Analysis of Human Memory B-Cell Subpopulations: IgD+ CD27+ B Cells Are Crucial in Secondary Immune Response by Producing High Affinity IgM. Clin Immunol (2003) 108(2):128–37. doi: 10.1016/S1521-6616(03)00092-5 12921759

[20] Liu W Meckel T Tolar P Sohn HW Pierce SK. Intrinsic Properties of Immunoglobulin IgG1 Isotype-Switched B Cell Receptors Promote Microclustering and the Initiation of Signaling. Immunity (2010) 32(6):778–89. doi: 10.1016/j.immuni.2010.06.006 PMC2904325 20620943

[21] Lutz J Dittmann K Bösl MR Winkler TH Wienands J Engels N. Reactivation of IgG-Switched Memory B Cells by BCR-Intrinsic Signal Amplification Promotes IgG Antibody Production. Nat Commun (2015) 6(1):1–9. doi: 10.1038/ncomms9575 PMC4633962 26815242

[22] Van Keimpema M Grüneberg LJ Mokry M Van Boxtel R Van Zelm MC Coffer P. The Forkhead Transcription Factor FOXP1 Represses Human Plasma Cell Differentiation. Blood (2015) 126(18):2098–109. doi: 10.1182/blood-2015-02-626176 PMC4626252 26289642

[23] Kometani K Nakagawa R Shinnakasu R Kaji T Rybouchkin A Moriyama S. Repression of the Transcription Factor Bach2 Contributes to Predisposition of IgG1 Memory B Cells Toward Plasma Cell Differentiation. Immunity (2013) 39(1):136–47. doi: 10.1016/j.immuni.2013.06.011 23850379

[24] Zuccarino-Catania GV Sadanand S Weisel FJ Tomayko MM Meng H Kleinstein SH. CD80 and PD-L2 Define Functionally Distinct Memory B Cell Subsets That are Independent of Antibody Isotype. Nat Immunol (2014) 15(7):631–7. doi: 10.1038/ni.2914 PMC4105703 24880458

[25] Martin SW Goodnow CC. Burst-Enhancing Role of the IgG Membrane Tail as a Molecular Determinant of Memory. Nat Immunol (2002) 3(2):182–8. doi: 10.1038/ni752 11812996

[26] Kato Y Abbott RK Freeman BL Haupt S Groschel B Silva M. Multifaceted Effects of Antigen Valency on B Cell Response Composition and Differentiation *In Vivo* . Immunity (2020) 53(3):548–563.e548. doi: 10.1016/j.immuni.2020.08.001 32857950 PMC7451196

[27] Pape KA Maul RW Dileepan T Paustian AS Gearhart PJ Jenkins MK. Naive B Cells With High-Avidity Germline-Encoded Antigen Receptors Produce Persistent IgM+ and Transient IgG+ Memory B Cells. Immunity (2018) 48(6):1135–43.e1134. doi: 10.1016/j.immuni.2018.04.019 29884459 PMC6052797

[28] Klasse P. How to Assess the Binding Strength of Antibodies Elicited by Vaccination Against HIV and Other Viruses. Expert Rev Vaccines (2016) 15(3):295–311. doi: 10.1586/14760584.2016.1128831 26641943 PMC4766047

[29] Alexander MR Ringe R Sanders RW Voss JE Moore JP. Klasse PJJJov. What do Chaotrope-Based Avidity Assays for Antibodies to HIV-1 Envelope Glycoproteins Measure? J Virol (2015) 89(11):5981–95. doi: 10.1128/JVI.00320-15 PMC4442429 25810537

[30] Cascino K Roederer M Liechti T. OMIP-068: High-Dimensional Characterization of Global and Antigen-Specific B Cells in Chronic Infection. Cytometry A (2020) 97(10):1037–43. doi: 10.1002/cyto.a.24204 PMC7581549 32741082

[31] Brooks JF Liu X Davies JM Wells JW Steptoe RJ. Tetramer-Based Identification of Naïve Antigen-Specific B Cells Within a Polyclonal Repertoire. Eur J Immunol (2018) 48(7):1251–4. doi: 10.1002/eji.201747447 29572817

[32] Paus D Phan TG Chan TD Gardam S Basten A Brink R. Antigen Recognition Strength Regulates the Choice Between Extrafollicular Plasma Cell and Germinal Center B Cell Differentiation. J Exp Med (2006) 203(4):1081–91. doi: 10.1084/jem.20060087 PMC2118299 16606676

[33] Phan TG Paus D Chan TD Turner ML Nutt SL Basten A. High Affinity Germinal Center B Cells are Actively Selected Into the Plasma Cell Compartment. J Exp Med (2006) 203(11):2419–24. doi: 10.1084/jem.20061254 PMC2118125 17030950

[34] Schamel WW Reth M. Monomeric and Oligomeric Complexes of the B Cell Antigen Receptor. Immunity (2000) 13(1):5–14. doi: 10.1016/S1074-7613(00)00003-0 10933390

[35] Tolar P Sohn HW Pierce SK. The Initiation of Antigen-Induced B Cell Antigen Receptor Signaling Viewed in Living Cells by Fluorescence Resonance Energy Transfer. Nat Immunol (2005) 6(11):1168–76. doi: 10.1038/ni1262 16200067

[36] Koutsakos M Wheatley AK Loh L Clemens EB Sant S Nüssing S. Circulating TFH Cells, Serological Memory, and Tissue Compartmentalization Shape Human Influenza-Specific B Cell Immunity. Sci Trans Med (2018) 10(428). doi: 10.1126/scitranslmed.aan8405 29444980

[37] Bentebibel S-E Khurana S Schmitt N Kurup P Mueller C Obermoser G. ICOS+ PD-1+ CXCR3+ T Follicular Helper Cells Contribute to the Generation of High-Avidity Antibodies Following Influenza Vaccination. Sci Rep (2016) 6(1):1–8. doi: 10.1038/srep26494 27231124 PMC4882544

[38] Bentebibel S-E Lopez S Obermoser G Schmitt N Mueller C Harrod C. Induction of ICOS+ CXCR3+ CXCR5+ TH Cells Correlates With Antibody Responses to Influenza Vaccination. Sci Trans Med (2013) 5(176):176ra132–176ra132. doi: 10.1126/scitranslmed.3005191 PMC3621097 23486778

[39] Herati RS Muselman A Vella L Bengsch B Parkhouse K Del Alcazar D. Successive Annual Influenza Vaccination Induces a Recurrent Oligoclonotypic Memory Response in Circulating T Follicular Helper Cells. Sci Immunol (2017) 2(8). doi: 10.1126/sciimmunol.aag2152 PMC5469419 28620653

[40] Lindgren G Ols S Liang F Thompson EA Lin A Hellgren F. Induction of Robust B Cell Responses After Influenza mRNA Vaccination Is Accompanied by Circulating Hemagglutinin-Specific ICOS+ PD-1+ CXCR3+ T Follicular Helper Cells. Front Immunol (2017) 8:1539. doi: 10.3389/fimmu.2017.01539 29181005 PMC5693886

[41] Abudulai LN Fernandez S Corscadden K Burrows SA Hunter M Tjiam MC. Production of IgG Antibodies to Pneumococcal Polysaccharides is Associated With Expansion of ICOS+ Circulating Memory T Follicular-Helper Cells Which is Impaired by HIV Infection. PloS One (2017) 12(5):e0176641. doi: 10.1371/journal.pone.0176641 28463977 PMC5413043

[42] Heit A Schmitz F Gerdts S Flach B Moore MS Perkins JA. Vaccination Establishes Clonal Relatives of Germinal Center T Cells in the Blood of Humans. J Exp Med (2017) 214(7):2139–52. doi: 10.1084/jem.20161794 PMC5502430 28637884

[43] Farooq F Beck K Paolino KM Phillips R Waters NC Regules JA. Circulating Follicular T Helper Cells and Cytokine Profile in Humans Following Vaccination With the rVSV-ZEBOV Ebola Vaccine. Sci Rep (2016) 6(1):1–9. doi: 10.1038/srep27944 27323685 PMC4914957

[44] Huber JE Ahlfeld J Scheck MK Zaucha M Witter K Lehmann L. Dynamic Changes in Circulating T Follicular Helper Cell Composition Predict Neutralising Antibody Responses After Yellow Fever Vaccination. Clin Trans Immunol (2020) 9(5):e1129. doi: 10.1002/cti2.1129 PMC7221214 32419947

[45] Brenna E Davydov AN Ladell K McLaren JE Bonaiuti P Metsger M. CD4+ T Follicular Helper Cells in Human Tonsils and Blood are Clonally Convergent But Divergent From non-Tfh CD4+ Cells. Cell Rep (2020) 30(1):137–52.e135. doi: 10.1016/j.celrep.2019.12.016 31914381 PMC7029615

[46] Ellebedy AH Jackson KJ Kissick HT Nakaya HI Davis CW Roskin KM. Defining Antigen-Specific Plasmablast and Memory B Cell Subsets in Human Blood After Viral Infection or Vaccination. Nat Immunol (2016) 17(10):1226. doi: 10.1038/ni.3533 27525369 PMC5054979

[47] Giesecke C Frölich D Reiter K Mei HE Wirries I Kuhly R. Tissue Distribution and Dependence of Responsiveness of Human Antigen-Specific Memory B Cells. J Immunol (2014) 192(7):3091–100. doi: 10.4049/jimmunol.1302783 24567530

[48] Lau D Lan LY-L Andrews SF Henry C Rojas KT Neu KE. Low CD21 Expression Defines a Population of Recent Germinal Center Graduates Primed for Plasma Cell Differentiation. Sci Immunol (2017) 2(7). doi: 10.1126/sciimmunol.aai8153 PMC5896567 28783670

[49] Portugal S Obeng-Adjei N Moir S Crompton PD Pierce SK. Atypical Memory B Cells in Human Chronic Infectious Diseases: An Interim Report. Cell Immunol (2017) 321:18–25. doi: 10.1016/j.cellimm.2017.07.003 28735813 PMC5732066

[50] Sutton HJ Aye R Idris AH Vistein R Nduati E Kai O. Atypical B Cells are Part of an Alternative Lineage of B Cells That Participates in Responses to Vaccination and Infection in Humans. Cell Rep (2021) 34(6):108684. 33567273 10.1016/j.celrep.2020.108684 PMC7873835

[51] Budeus B de Reynoso SS Przekopowitz M Hoffmann D Seifert M Küppers R. Complexity of the Human Memory B-Cell Compartment is Determined by the Versatility of Clonal Diversification in Germinal Centers. Proc Natl Acad Sci (2015) 112(38):E5281–9. doi: 10.1073/pnas.1511270112 PMC4586852 26324941

[52] Gitlin AD von Boehmer L Gazumyan A Shulman Z Oliveira TY Nussenzweig MC. Independent Roles of Switching and Hypermutation in the Development and Persistence of B Lymphocyte Memory. Immunity (2016) 44(4):769–81. doi: 10.1016/j.immuni.2016.01.011 PMC4838502 26944202

[53] Roco JA Mesin L Binder SC Nefzger C Gonzalez-Figueroa P Canete PF. Class-Switch Recombination Occurs Infrequently in Germinal Centers. Immunity (2019) 51(2):337–50.e337. doi: 10.1016/j.immuni.2019.07.001 31375460 PMC6914312

[54] Locci M Havenar-Daughton C Landais E Wu J Kroenke MA Arlehamn CL. Human Circulating PD-1+ CXCR3– CXCR5+ Memory Tfh Cells Are Highly Functional and Correlate With Broadly Neutralizing HIV Antibody Responses. Immunity (2013) 39(4):758–69. doi: 10.1016/j.immuni.2013.08.031 PMC3996844 24035365

[55] Morita R Schmitt N Bentebibel S-E Ranganathan R Bourdery L Zurawski G. Human Blood CXCR5+ CD4+ T Cells are Counterparts of T Follicular Cells and Contain Specific Subsets That Differentially Support Antibody Secretion. Immunity (2011) 34(1):108–21. doi: 10.1016/j.immuni.2010.12.012 PMC3046815 21215658

[56] Bowyer G Grobbelaar A Rampling T Venkatraman N Morelle D Ballou RW. CXCR3+ T Follicular Helper Cells Induced by Co-Administration of RTS, S/AS01B and Viral-Vectored Vaccines Are Associated With Reduced Immunogenicity and Efficacy Against Malaria. Front Immunol (2018) 9:1660. doi: 10.3389/fimmu.2018.01660 30090099 PMC6068239

[57] Amanna IJ Carlson NE Slifka MK. Duration of Humoral Immunity to Common Viral and Vaccine Antigens. New Engl J Med (2007) 357(19):1903–15. doi: 10.1056/NEJMoa066092 17989383

[58] Plotkin SA. Correlates of Protection Induced by Vaccination. Clin Vaccine Immunol (2010) 17(7):1055–65. doi: 10.1128/CVI.00131-10 PMC2897268 20463105

[59] Reiss S Baxter AE Cirelli KM Dan JM Morou A Daigneault A. Comparative Analysis of Activation Induced Marker (AIM) Assays for Sensitive Identification of Antigen-Specific CD4 T Cells. PloS One (2017) 12(10):e0186998. doi: 10.1371/journal.pone.0186998 29065175 PMC5655442

[60] Havenar-Daughton C Reiss SM Carnathan DG Wu JE Kendric K de la Peña AT. Cytokine-Independent Detection of Antigen-Specific Germinal Center T Follicular Helper Cells in Immunized Nonhuman Primates Using a Live Cell Activation-Induced Marker Technique. J Immunol (2016) 197(3):994–1002. doi: 10.4049/jimmunol.1600320 27335502 PMC4955744

[61] National Statement on Ethical Conduct in Human Research: 2007 (Updated 2018). In: National Health and Medical Research Council. Commonwealth of Australia Canberra.

[62] Mahnke Y Chattopadhyay P Roederer M. Publication of Optimized Multicolor Immunofluorescence Panels. Cytometry A (2010) 77(9):814–8. doi: 10.1002/cyto.a.20916 20722004

[63] Chen H Lau MC Wong MT Newell EW Poidinger M Chen J. Cytofkit: A Bioconductor Package for an Integrated Mass Cytometry Data Analysis Pipeline. PloS Comput Biol (2016) 12(9):e1005112. doi: 10.1371/journal.pcbi.1005112 27662185 PMC5035035

[64] Dimitrov JD Lacroix-Desmazes S Kaveri SV. Important Parameters for Evaluation of Antibody Avidity by Immunosorbent Assay. Analyt Biochem (2011) 418(1):149–51. doi: 10.1016/j.ab.2011.07.007 21803020

[65] Olsson J Johansson J Honkala E Blomqvist B Kok E Weidung B. Urea Dilution of Serum for Reproducible Anti-HSV1 IgG Avidity Index. BMC Infect Dis (2019) 19(1):164. doi: 10.1186/s12879-019-3769-x 30764767 PMC6376645

