# Supplementary material for: Characterising the Phenotypic Diversity of Antigen-Specific Memory B Cells Before and After Vaccination
Source: Front Immunol. 2021 Sep 28;12:738123. doi: 10.3389/fimmu.2021.738123 (PMC8505969; doi:10.3389/fimmu.2021.738123)
Supplement: Supplementary file 1 [file Table_1.docx]

Supplementary table 1. Fluorescent staining reagents used for the assessment of TTd-specific B cells and cT_FH_ cells by flow cytometry. [Panel 1 = Phenotypic characterisation of cT_FH_, Panel 2 = Phenotypic characterisation of TTd-specific B cells, Panel 3 = CD79b-normalised TT binding to B cells]

| **Item** | **Clone** | **Manufacturer (Item#)** | **Panel** |
| --- | --- | --- | --- |
| CCR6 BV510 | 11A9 | BD Horizon™ 563241 | 1 |
| CD14 PerCP-Cy5.5 | M5E2 | BD Pharmingen 550787 | 2, 3 |
| CD20 BV510 | MS4A1 | BD Horizon™ 563067 | 2, 3 |
| CD27 BB515 | M-T271 | BD Horizon™ 564642 | 1, 2, 3 |
| CD3 PerCP-Cy5.5 | SK7 | BD Biosciences 332771 | 2, 3 |
| CD3 AF700 | UCHT-1 | BD Horizon™ 557943 | 1 |
| CD38 APC-H7 | HB7 | BD Biosciences 656646 | 2, 3 |
| CD4 APC-H7 | RPA-T4 | BD Biosciences 560158 | 1 |
| CD45RA PerCP-Cy5.5 | HI-100 | BD Pharmingen™ 563429 | 1 |
| CD56 BB700 | NCAM16.2 | BD Horizon™ 566573 | 2, 3 |
| CD79b APC-R700 | SN8 | BD Horizon™ 657702 | 3 |
| CXCR3 PE-Cy7 | 1C6 | BD Pharmingen™ 560831 | 1 |
| CXCR5 BV421 | RF8B2 | BD Horizon™ 562747 | 1 |
| ICOS PE | DX29 | BD Pharmingen™ 557802 | 1 |
| IgA PE | Goat pAb | Southern Biotech 2050-09 | 2 |
| IgD AF700 | IA6-2 | BD Pharmingen 561302 | 2 |
| IgD PE-Cy7 | IA6-2 | BD Pharmingen™ 561314 | 3 |
| IgG PE-Cy7 | G18-145 | BD Pharmingen 561298 | 2 |
| IgG PE | G18-145 | BD Pharmingen™ 555787 | 3 |
| IgM PE-CF594 | G20-127 | BD Horizon™ 562539 | 2, 3 |
| PD-1 APC | MIH4 | BD Pharmingen™ 558694 | 1 |
| BV421 streptavidin | n/a | BD Horizon™ 563259 | 2, 3 |
| AF647 streptavidin | n/a | BioLegend 405237 | 2, 3 |
| FVS 575V | n/a | BD Horizon™ 565694 | 1, 2, 3 |
